# Advancing paediatric robotic surgery: building the foundation with databases

**DOI:** 10.1007/s11701-026-03774-y

**Published:** 2026-08-12

**Authors:** Calvin George Marangoly, Thomas Blanc, Mohan S. Gundeti, Craig A. Peters, Chris Kimber, Kiarash Taghavi

**Affiliations:** 1 Monash School of Medicine, Monash University, Melbourne, Australia; 2 Université Paris Cité, F-75006 Paris, France; 3 Service de Chirurgie viscérale, urologie et transplantation pédiatrique, AP-HP, Hôpital Necker-Enfants malades, F-75015 Paris, France; 4 Section of Urology, Department of Surgery, University of Chicago, Chicago, IL USA; 5 Department of Urology, Division of Pediatric Urology, University of Texas Southwestern/Children’s Medical Center Dallas, Dallas, TX USA; 6 Department of Paediatric Urology, Monash Children’s Hospital, Monash Health, 246 Clayton Rd, VIC 3168 Clayton, Melbourne, Australia; 7 Department of Paediatrics, Monash University, Melbourne, Australia

**Keywords:** Robotic surgery, Pediatric surgery, Pediatric urology, Database, Health informatics, Data points

## Abstract

This study explores implementation of robotic surgery in children emphasising the critical role of data informatics. Development of purpose-built multi-centre databases and collaborative prospective studies will facilitate ongoing expansion, establishment of a robust evidence-base, and ultimately enhance surgical outcomes. We conducted interviews with leading experts in paediatric robotic surgery—Prof. Thomas Blanc, Prof. Mohan Gundeti, and Prof. Craig Peters. Their insights were synthesised to develop a database framework, which will serve dual purposes: quality assurance and facilitating research initiatives. Various data management platforms were evaluated, including Microsoft Excel, Research Electronic Data Capture (REDCap), as well as custom-built solutions, each demonstrating distinct advantages in usability, accessibility, security, and cost-effectiveness. Data points were identified through review of extensive clinical experience, collaborative brainstorming sessions and forward-thinking strategies. Based on expert consultation, a suggested REDCap database was developed for future use. The interviewed experts unanimously emphasised the importance of dedicated personnel and shared objectives in ensuring the future-proofing, sustainability and overall effectiveness of clinical research databases. This study highlights the imperative for interdisciplinary collaboration and institution of data informatics at the point of establishment of paediatric robotic programs.

**Supplementary Information:**

The online version contains supplementary material available at 10.1007/s11701-026-03774-y.

## Introduction

Robotic surgery has emerged as a new frontier in minimally invasive surgery (MIS), offering significant benefits over open techniques and incremental benefits over laparoscopic approaches, including enhanced visualisation, precision and recovery [1]. The predominant application of robotic surgery at this stage has been in adult urology. Challenges related to instrumentation, anatomical considerations, and barriers to implementation have limited adoption in paediatric populations to date [2]. Nonetheless, application of robotic systems within paediatric surgery is expanding [3]. Because robot-assisted surgery in children is still emerging in many regions globally, there is a timely and critical need for standardised data informatics to ensure interoperability. Providing a framework for quality assurance, outcome monitoring, and multi-centre collaboration is essential for centres early in this journey to ensure patient safety and establishment of a robust evidence base.

Given the substantial investment required in establishing a robotic surgical program, the development of a robust evidence base is imperative. Health informatics plays a pivotal role in advancing evidence-based medicine [4] by facilitating systematic collection, organisation and analysis of data for clinical research. Databases are broadly classified into two categories: administrative and clinical. Administrative databases primarily contain billing and healthcare claims data, including diagnoses and treatments coded using systems such as the International Classification of Diseases, Tenth Revision (ICD-10) [5, 6]. These databases provide extensive datasets over prolonged periods and are generally cost-effective [5], making them accessible to healthcare institutions with minimal effort and financial outlay. However, administrative databases are limited as they often lack detailed clinical and operational information [6]. Additionally, coding biases may arise for financial reasons, potentially impacting the accuracy of the data [7].

In contrast, clinical databases are crucial for surgical research, designed specifically to address targeted clinical questions and hypothesis development [5]. They can vary significantly in scope and breadth, ranging from single-institutional databases to multi-institutional and international collaborative databases. Single-institution databases typically offer granularity and detailed insights, while larger multi-institutional databases reduce selection bias and are often better powered particularly for rare conditions, but may entail higher costs [5, 6]. Each type of database serves a distinct purpose in advancing clinical knowledge and enhancing patient care. For example, in the broader paediatric context, established clinical databases such as the American College of Surgeons National Surgical Quality Improvement Program Pediatric (ACS NSQIP-P) have been highly effective in tracking surgical outcomes and quality metrics [5], though they lack robot-specific granularity.

Current clinical databases in adult urology serve as vital resources for capturing patient outcomes and informing best practices [5]. For instance, the Clinical Research Office of the Endourological Society (CROES) Ureteroscopy Global Study exemplifies how international multi-centre data collection can enhance understanding of intraoperative and postoperative factors, demonstrating safety and efficacy of stone management [8]. Similarly, large-scale databases such as the Robotic Surgery for Upper Tract Urothelial Cancer Study (ROBUUST) 2.0 [9] and European Association of Urology (EAU) Robotic Urology Section (ERUS) Scientific Working Group [10] have provided invaluable insights into the effectiveness of robotic procedures in adult populations, particularly through the analysis of survival rates and length of stay.

However, while the clinical insights generated by adult databases are widely published, there is a distinct lack of literature describing the methodological frameworks required to actually structure them. Furthermore, the development of paediatric robotic databases requires unique considerations over and above standard surgical databases. Unlike adult cohorts, paediatric quality of life and postoperative outcomes frequently rely on surrogate reporting by parents or guardians, necessitating the use of specialised, validated tools like the Child Health Utility 9D (CHU9D) index. Combined with metrics specific to the robotic system in smaller anatomical workspaces (e.g. docking time, conversions, robot-specific complications and morbidity, etc.), there remains a distinct lack of guidelines or frameworks for the establishment of paediatric robotic databases. This study aims to synthesise insights from pioneering paediatric robotic surgeons who have successfully implemented paediatric robotic programs across various institutions internationally, thereby addressing existing gaps and providing the platform for a more robust evidence base in paediatric robotic surgery.

## Methods

TB, MG and CP were selected and contacted by KT for interviews based on their pioneering role in establishing paediatric robotic surgery programs in major centres, specifically Paris (2016), Chicago (2007), and Boston (2002). Each expert employed distinct methodologies for creating, maintaining and expanding their clinical databases.

Three separate 30-minute semi-structured interviews were facilitated by CGM and conducted via Zoom with the objective of understanding each interviewee’s reflections on constructing paediatric robotic surgery databases. KT and CGM brainstormed beforehand the broad topics that each interview would cover, including advantages and disadvantages of the platforms utilised, the selection of data points, notable initiatives implemented, and any regrets experienced throughout the process (see Online Resource 1 for the interview questions).

All interviews were recorded and stored in Zoom’s cloud software under CGM’s account due to file size constraints. Transcriptions of each interview were generated via Zoom’s in-built functionality and downloaded onto CGM’s personal hard drive to facilitate summarisation in a Microsoft Word document. Given the focused cohort of three experts, formal thematic analysis was not performed; rather, all comments and unique insights from each expert were consolidated in full. In synthesising the participants’ current data collection schemes into a unified model, specific consideration was given to generalisability, usability, and research utility. While no follow-up interviews were necessary, the final proposed database proforma was sent to all three interviewees for review, ratification, and agreement.

## Results

The interviews yielded several core strategic insights regarding the establishment, scalability, and long-term maintenance of surgical databases. These foundational lessons are summarised in Table 1. The subsequent sections expand upon these insights by detailing the experts’ practical experiences across four key domains: digital platforms, patient data collection, complications/outcomes, and the human element. Application of these insights to the proposed database proforma provides a purpose-built, foundational step towards greater consistency in paediatric robotic surgical research.

**Table 1:** Strategic Insights and Lessons Learnt from Expert Interviews

Strategic Insight	Source	Context and Lesson Learnt
Anticipating scalability	TB, MG	Both initially utilised standalone spreadsheets but recognised the necessity of transitioning to secure, collaborative platforms (e.g. REDCap, CleanWeb) to support multi-centre expansion
System interoperability	TB, MG	To prevent data silos, databases should integrate with existing electronic health record (EHR) systems. TB demonstrated this by syncing CleanWeb directly with his hospital’s EHR, a vital feature MG also endorsed for future databases
Comprehensive data capture	MG, CP, TB	Establishing a broad base of variables is crucial. MG advised capturing as many variables as possible initially, while CP emphasised the inclusion of subjective, patient-centred metrics and novel biological markers
Early (prospective) data capture	CP, TB, MG	Retrospective data entry is costly, time-consuming, and introduces bias. CP explicitly cited a regret of not keeping a highly detailed database from the very inception of the robotic program
Managing the administrative burden	MG, TB	Data-entry fatigue is a primary barrier. Sustained success requires delegating tasks to dedicated, long-term personnel (e.g. research nurses or data managers)

## Platforms

Initially, all interviewees utilised Microsoft Excel for their data management needs. The choice of platform for expansion was largely influenced by the network of institutions involved. CP continued to use Excel for his single-institution database, while MG consulted with colleagues and hospital executives and transitioned to REDCap to facilitate multi-institutional collaboration. TB, in conjunction with software engineers and after initially collaborating with various paediatric surgical subspecialties, developed a custom-built platform (“CleanWeb”), which is integrated with the Assistance Publique-Hôpitaux de Paris (AP-HP) electronic health record (EHR) system [11] and was later expanded to include adult robotic surgery. Data privacy was maintained by institutional review boards, with access restricted to authorised researchers via institutional IT infrastructure.

### Microsoft Excel

Excel is recognised for its user-friendly interface, allowing for straightforward data entry and analysis without the need for dedicated IT personnel. Data security is maintained through the use of hospital-authorised devices. However, the manual data entry can be time-consuming and is susceptible to errors [12], particularly as datasets grow. Although research assistants can assist with data entry, this incurs additional costs. Furthermore, collaboration across different institutions presents challenges when relying solely on Excel as data security concerns would prevent sharing of Microsoft documents between institutions. Similar platforms such as Google Sheets allow for enhanced collaboration and seamless real-time database updates but also present a data security risk as relevant files would not be stored on hospital-authorised servers or hard drives.

### REDCap

REDCap is a web-based application designed for customisable data collection and storage [13] that is accessible without requiring programming skills. It includes data quality and validation tools that help mitigate the potential for errors [14]. The REDCap platform offers greater security than Excel and facilitates multi-centre collaboration; however, access requires institutional affiliation with the REDCap consortium, and a REDCap administrator is necessary for effective management [13, 14].

### Custom-built solutions

Custom-built solutions allow for comprehensive database customisation and provide control over data security through existing IT infrastructure. Integration with the EHR streamlines data collection and minimises clinician workload [11]. However, implementing a custom solution is resource-intensive, requiring collaboration among software engineers, data managers and clinicians. Some EHR providers, notably Epic Systems, offer integrated databases that draw on data collected from participating institutions [15], allowing for a convenient yet costly alternative.

### Clinical data warehouses (CDWs)

Clinical data warehouses collate data from a variety of sources [16], enhancing the breadth of available information. For instance, the French Système National des Données de Danté (SNDS) encompasses data from 99% of the population [17]. However, these databases often lack detailed clinical information and typically require software engineers to facilitate data access.

In summary, the examination of various platforms underscores the importance of surgeons selecting the right solution based on institutional needs, available resources, and the desired level of data granularity and security.

## Patient Data Collection

The interviewees employed a similar methodology for determining relevant data points for their databases. Given the absence of established paediatric robotic databases, they each constructed their database by engaging in explorative sessions with their colleagues to identify potential data points, analysing existing literature, and incorporating their clinical experience. The variables ultimately identified by each interviewee shared significant overlap despite conducting this process independently and practicing in different regions around the world, demonstrating the generalisability of a well-constructed database.

**Fig. 1:**
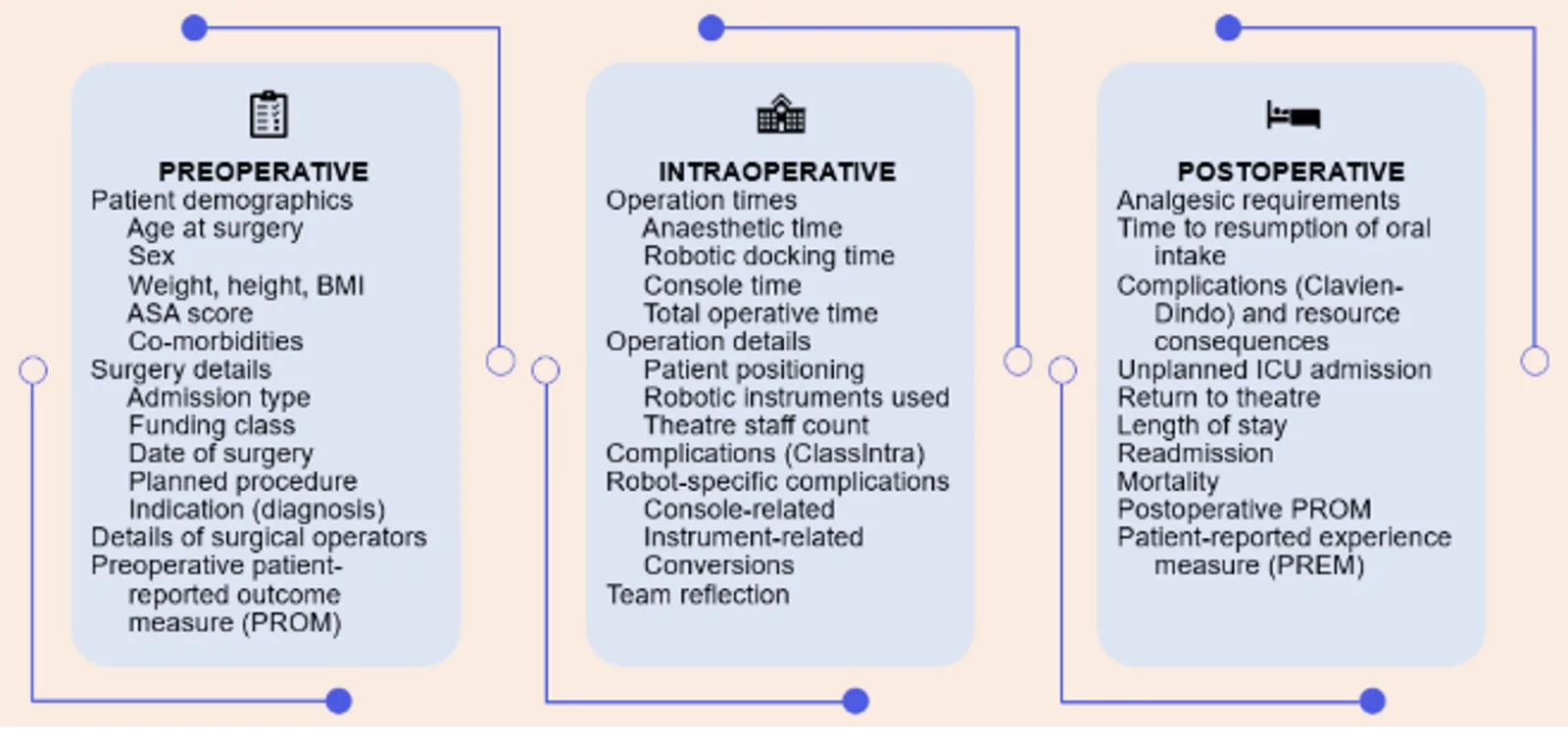
Data points to include in a paediatric robotic surgery database

MG emphasised the importance of identifying as many variables of interest as possible from the outset, as revising databases later can be labour-intensive. Data points should align with those of existing surgical databases while incorporating robot-specific and paediatric-specific metrics (see Fig. 1).

Many databases utilise alphanumeric coding systems, like ICD-10 and Current Procedural Terminology. However, these systems can fail to capture nuanced details as they are primarily mono-hierarchical, potentially leading to misclassification of diseases [18].

Key operating room metrics, including docking time, console time, and total operative time, are critical in understanding operating room efficiency and surgeon learning curves. While improving operative times may not be a principal objective of robot-assisted surgery, for institutions or programs where operational workflow is a key motivation, measuring these times provides valuable objective data. Although initially operative times may be longer, they tend to decrease with increased experience over approximately the first 30 cases [19, 20]. The learning curve of any new technology can be a barrier to its adoption. However, because the learning curve for robot-assisted surgery is often considered faster than that of laparoscopy, using databases to understand the particular learning curve for various robotic interventions may offer evidence to support broader adoption. Nevertheless, variability in definitions of the learning curve and the metrics used for outcomes assessment complicates this issue [21]. Accredited standardised training programs for robotic surgery are not yet widely established [22], which results in operative practices that are primarily institutional based, consequently exacerbating this heterogeneity [23].

Console time may be further subdivided into components related to specific stages of procedures, such as dissection and anastomosis during pyeloplasty. Recording key operating room metrics allows robotic surgeons to measure their progress objectively and prompt reflective practise. Furthermore, it allows broader evaluation regarding the feasibility of robot-assisted surgery in various applications, efficacy and surgical outcomes, and a surgeon’s learning curve and training output.

## Complications and Outcomes

Post-operative complications are classified using the widely accepted Clavien-Dindo classification [24], which has been validated across various surgical subspecialties [25]. Recently, intraoperative complications have been characterised using an organisational system called ClassIntra, which shares intentional similarities with the Clavien-Dindo system and has also been validated in multiple subspecialties [26]. Robot-related complications are generally classified into two categories: issues with the robot or console (e.g. software issues, video loss) and robotic arms (e.g. collisions, component malfunctions) [27]. Measuring conversions to open surgery is also critical and high rates may be attributable to poor case selection [1], as it is advisable to choose simpler cases initially to aid the learning curve [2]. Furthermore, while this generic framework focuses on core perioperative and short-term metrics, establishing these large, multi-centre cohorts provides the essential foundation for subsequent thorough interrogation—either prospectively or retrospectively—of procedure-specific long-term outcomes, which remain a crucial yet frequently overlooked aspect of paediatric surgical research.

CP believes understanding the benefits of robot-assisted surgery necessitates more than the collection of objective variables. The advantages for both patients and surgeons can be difficult to quantify in absolute terms. Therefore, researchers should consider incorporating patient-centred measures, such as family perceptions, into the database. Patient-reported outcome measures and experience measures (PROMs, PREMs) are two types of questionnaires used to evaluate healthcare quality from the perspective of the patient. PROMs measure how effectively treatment has improved the patient’s quality of life and so is useful to record before and after surgery, whereas PREMs assess the patient’s overall healthcare experience with the hospital system and treating team rather than just symptomatic improvement [28, 29]. In the future, surgeon perspectives and experience measures should also be incorporated into these databases, although this field is still in its infancy. Aspects such as the surgeon’s cognitive load, ergonomics, physical risk of injury, ability to respond to operative variations, and overall satisfaction with the quality of the procedure are crucial metrics, though standardised and validated tools to routinely capture these in clinical registries have yet to be fully developed.

For the implementation of any novel technology, it is also vital to assess costs associated with paediatric robotics programs. Capturing administrative details including the patient’s admission type and funding class allows data to be linked to hospital financing. Recording theatre staffing counts and the quantity and type of robotic instruments used allows for intraoperative expenses to be calculated. However, a more comprehensive analysis of the robot’s overall economic impact on institutional costs would require consideration of downstream costs and cost-savings, such as reductions in length of stay [30].

MG cautions that databases must be adaptable as new information and research questions arise to ensure that both the program and the database remain contemporary over time.

## The Human Element

The drive and vision of individuals are fundamental to the success of any project, particularly in the establishment of clinical databases. The interviewees emphasised the significance of human factors as essential in this process.

TB highlighted the importance of intra- and inter-institutional communication and teamwork to ensure the success of large-scale databases. He advocated for a strategic and stepwise approach to centre inclusion and database expansion, emphasising the importance of aligning goals and resources.

MG highlighted the necessity of having long-term and stable research assistants to ensure continuity and facilitate sustainable long-term outcomes. Their presence fosters a deeper understanding of the evolving needs of the database and contributes to its ongoing refinement.

CP underscored the value of conducting debriefing sessions following each robotic procedure. These discussions are vital for analysing the variables that have been collected and for identifying areas where improvements can be made. Maintaining consistent teams is essential for building collective experience, which ultimately enhances the overall effectiveness and reliability of the database.

In summary, the human element—comprised of communication, teamwork, collaboration, consistency and commitment to continuous improvement—is pivotal in fostering a successful paediatric robotic surgery database.

## Future Directions: Proposed Database Proforma

Through sequential expert consultation, we developed a prospective database which has already been applied to paediatric robotic surgeries across multiple institutions in Australia. REDCap was selected as the platform of choice given the ease of collaboration and our academic institution’s pre-existing infrastructure relating to the REDCap platform. The final structure and data points selected arose through an iterative process of brainstorming and real-world trials. Additional insight was provided by a research fellow specialising in health economics, who suggested the inclusion of specific variables that would allow for more thorough health economic analyses to be conducted using the database. The entire database was reviewed by a research software and systems engineer and REDCap specialist, who suggested modifications to the layout, branching logic and option coding of various questions within the REDCap ecosystem to optimise the experience for both the user and future data analysts.

The database is grouped into preoperative, intraoperative and postoperative parts and separated into REDCap instruments for ease of accessibility. Preoperative variables include non-identifying patient demographics (e.g. age, sex, comorbidities), surgery details (including admission type and funding class), and information about the surgeons’ experience with robotic and non-robotic operations (see Online Resource 2). The preoperative PROM used in this database is the Child Health Utility 9D (CHU9D) index, selected because it is comprehensive, measuring nine dimensions each at five levels, and has been validated for use in children aged 4 to 18 years, with parents completing the index on behalf of their children [31] (see Online Resource 3). Intraoperative data includes standard surgical research variables (e.g. operating times, intraoperative complications) along with robot-specific data (e.g. robotic console time, number and types of robotic instruments used) and a space to document reflections of the operating team (see Online Resource 4). The postoperative aspect of the database is split into three instruments for practicality, one detailing typical metrics (e.g. analgesic requirements, length of stay, postoperative complications and their resource consequences; see Online Resource 5), one being the postoperative CHU9D questionnaire (see Online Resource 6), and the other being a national standard PREM called the Australian Hospital Patient Experience Questionnaire Set (see Online Resource 7).

Anonymity of data entered into REDCap is achieved by stripping data of identifying information. However, an Excel spreadsheet located on our institution’s local servers contains identifying demographics of each case and is linked to each record in the database by REDCap’s Record ID. This allows surgeons undertaking case-based analysis the ability to find specific information while ensuring adherence to patient confidentiality as required by an internal ethics committee.

## Conclusion

As the adoption of paediatric robotic surgery continues to expand, the establishment of comprehensive and standardised clinical databases remains essential to evaluate ongoing safety, efficacy and clinical utility. The insights provided by TB, MG, and CP were invaluable in shaping this analysis of the critical features necessary for a paediatric robotic surgery database.

A clinical, rather than administrative, database is likely to be the most effective tool, with the choice of platform being influenced by factors such as size, user-friendliness, and cost. It should encompass a comprehensive set of variables, including standard surgical data points as well as robot-specific metrics. Through expert insight, we created a sample REDCap database for use in paediatric robotic surgery.

Furthermore, the success of the program and the associated research is contingent upon teamwork, incremental development, and a strong culture of safety. By prioritising these elements, stakeholders can cultivate an environment conducive to advancing paediatric robotic surgery and enhancing patient outcomes.

## Supplementary Information

Supplementary Material 1

Supplementary Material 2

Supplementary Material 3

Supplementary Material 4

Supplementary Material 5

Supplementary Material 6

Supplementary Material 7

## Data Availability

No datasets were generated or analysed during the current study.
