# Supplementary material for: Advancing paediatric robotic surgery: building the foundation with databases
Source: J Robot Surg. 2026 Aug 12;20(1):832. doi: 10.1007/s11701-026-03774-y (PMC13468980; doi:10.1007/s11701-026-03774-y)
Supplement: Supplementary file 1 [file 11701_2026_3774_MOESM1_ESM.pdf]

Online Resource 1

Interview Questions

Advancing Paediatric Robotic Surgery: Building the Foundation with Databases

Journal of Robotic Surgery

Calvin George Marangoly, Thomas Blanc, Mohan S. Gundeti, Craig A. Peters, Chris Kimber, Kiarash Taghavi

Corresponding author: Kiarash Taghavi, Department of Paediatric Urology, Monash Children's Hospital. Email: kiarash.taghavi@monash.edu

Interview with Prof. Thomas Blanc

- Could you tell me a bit about yourself and your experiences with paediatric robotic surgery?
- Prior to creating your paediatric robotic surgical database, did you have any experience with other robotic surgical databases? Do hospitals in France have experience establishing similar databases?
- What platform did you begin with? As you expanded, did you need to change platforms, and if so, what did platform(s) did you change to?
- How did you expand your database from being single institution to multi-institution? Was expanding a smooth process?
- Can you explain in more detail the SNDS database in France and how researchers extract data from it?
- How much does it cost for a researcher to access your database?
- How did you identify what robot-specific data points to collect? Which variables do you consider crucial to include?
- Have you ever found that you needed to edit the database to include more variables?
- Since many subspecialties were consulted during the process of database creation, were there many differences that needed to be ironed out?
- Is the process of data entry in your database cumbersome for clinicians? Did you need dedicated personnel to manage your database as it expanded?
- What processes are in place to ensure the security and privacy of the data collected?
- On reflection of your journey towards creating a paediatric robotic surgical database, what was a very valuable or crucial action you took and what would you do differently?

Interview with Prof. Mohan Gundeti

- Could you tell me a bit about yourself and your experiences with paediatric robotic surgery?
- Across your various positions internationally, what database platforms did you use? How did your database expand over time and who helped you along that journey?
- Did the financial burden of accessing US national surgical databases incentivise you to create your own database?
- How did you identify what robot-specific data points to collect? Which variables do you consider crucial to include?
- Is the process of data entry in your database cumbersome for clinicians? Did you need dedicated personnel to manage your database as it expanded?
- What processes are in place to ensure the security and privacy of the data collected?
- On reflection of your journey towards creating a paediatric robotic surgical database, what was a very valuable or crucial action you took and what would you do differently?

Interview with Prof. Craig Peters

- Could you tell me a bit about yourself and your experiences with paediatric robotic surgery?
- What database platform did you use? How was it managed?
- How did you identify what robot-specific data points to collect? Which variables do you consider crucial to include?
- Is the process of data entry in your database cumbersome for clinicians? Did you need dedicated personnel to manage your database as it expanded?
- What processes are in place to ensure the security and privacy of the data collected?
- On reflection of your journey towards creating a paediatric robotic surgical database, what was a very valuable or crucial action you took and what would you do differently?
