# Supplementary material for: Advancing paediatric robotic surgery: building the foundation with databases
Source: J Robot Surg. 2026 Aug 12;20(1):832. doi: 10.1007/s11701-026-03774-y (PMC13468980; doi:10.1007/s11701-026-03774-y)
Supplement: Supplementary file 2 [file 11701_2026_3774_MOESM2_ESM.pdf]

Online Resource 2

REDCap Database Proforma
Part 1.1 - Preoperative Variables

Advancing Paediatric Robotic Surgery: Building the Foundation with Databases

Journal of Robotic Surgery

Calvin George Marangoly, Thomas Blanc, Mohan S. Gundeti, Craig A. Peters, Chris Kimber, Kiarash Taghavi

Corresponding author: Kiarash Taghavi, Department of Paediatric Urology, Monash Children's Hospital. Email: kiarash.taghavi@monash.edu

Preoperative Variables

Please complete the survey below.

Thank you!

Patient Details

Study case number	__________________________________
Age of patient at surgery	__________________________________
Sex	◯ Male<br>◯ Female<br>◯ Another term<br>◯ Not stated or inadequately described
Another term for sex	__________________________________
Weight (kg)	__________________________________
Height (m)	__________________________________
BMI	__________________________________
ASA score	◯ ASA I<br>◯ ASA II<br>◯ ASA III<br>◯ ASA IV<br>◯ ASA V
Co-morbidities	____

Surgery Details

Planned admission type	◯ Elective day surgery<br>◯ Elective inpatient surgery<br>◯ Emergency
Funding class	◯ Public<br>◯ Private
Date of surgery	__________________________________
Planned procedure	◯ Pyeloplasty<br>◯ Nephrectomy<br>◯ Heminephrectomy<br>◯ Ureteric reimplantation<br>◯ Cholecystectomy<br>◯ Fundoplication<br>◯ Other
Other procedure	__________________________________
Indication	◯ PUJ obstruction<br>◯ Non-functioning kidney<br>◯ Pre-transplant<br>◯ Ectopic upper pole<br>◯ Non-functioning moiety<br>◯ VU reflex<br>◯ VU obstruction<br>◯ Cholelithiasis<br>◯ GORD<br>◯ Other
Other indication	__________________________________

Surgeon Details

Number of consultant robotic operators	◯ 1<br>◯ 2
Name of consultant operating surgeon 1	__________________________
Years of post-fellowship non-robotic surgical experience	__________________________
Years of post-fellowship robotic surgical experience	__________________________
Number of robotic operations completed as primary operator	__________________________
Name of consultant operating surgeon 2	__________________________
Years of post-fellowship non-robotic surgical experience	__________________________
Years of post-fellowship robotic surgical experience	__________________________
Number of robotic operations completed as primary operator	__________________________
