# Supplementary material for: Advancing paediatric robotic surgery: building the foundation with databases
Source: J Robot Surg. 2026 Aug 12;20(1):832. doi: 10.1007/s11701-026-03774-y (PMC13468980; doi:10.1007/s11701-026-03774-y)
Supplement: Supplementary file 4 [file 11701_2026_3774_MOESM4_ESM.pdf]

Online Resource 4

REDCap Database Proforma
Part 2 - Intraoperative Variables

Advancing Paediatric Robotic Surgery: Building the Foundation with Databases

Journal of Robotic Surgery

Calvin George Marangoly, Thomas Blanc, Mohan S. Gundeti, Craig A. Peters, Chris Kimber, Kiarash Taghavi

Corresponding author: Kiarash Taghavi, Department of Paediatric Urology, Monash Children's Hospital. Email: kiarash.taghavi@monash.edu

Intraoperative Variables

Please complete the survey below.

Thank you!

Operation Times

Time patient enters operating room __________________________________

Anaesthetic induction start time __________________________________

Skin incision time __________________________________

Anaesthetic time __________________________________

Time robotic arms are docked __________________________________

Robotic docking time __________________________________

Time robotic arms are undocked __________________________________

Robotic console time __________________________________

Skin closure time __________________________________

Total operative time __________________________________

Operation Details

Patient position	◯ Supine<br>◯ Prone<br>◯ Left lateral decubitus (right side up)<br>◯ Right lateral decubitus (left side up)<br>◯ Other
Other position	__________________________________
Route(s) of administration for local anaesthetic	☐ Subcutaneous injection<br>☐ Peripheral nerve block<br>☐ Nerve plexus block<br>☐ Intrathecal administration<br>☐ Epidural administration<br>☐ Other
Other route of administration	__________________________________
Estimated blood loss (mL)	__________________________________
Total number of robotic arms used	◯ 1<br>◯ 2<br>◯ 3<br>◯ 4
Was a laparoscopic assistant port used?	◯ Yes<br>◯ No

Type and Quantity of Robotic Instruments (Item Code) - Opened and USED

	1	2	3	4	5
5mm curved scissors (478057)	○	○	○	○	○
5mm permanent cautery hook (478090)	○	○	○	○	○
5mm suction irrigator (478054)	○	○	○	○	○
5mm Maryland bipolar forceps (478080)	○	○	○	○	○
5mm fenestrated bipolar forceps (478093)	○	○	○	○	○
5mm Cadiere forceps/grasper (478055)	○	○	○	○	○
5mm fundus grasper (478058)	○	○	○	○	○
5mm crocodile grasper (478059)	○	○	○	○	○
5mm curved needle driver (478088)	○	○	○	○	○
5mm wristed needle driver (478115)	○	○	○	○	○
5mm Maryland dissector (478050)	○	○	○	○	○
5mm medium-large clip applier (478053)	○	○	○	○	○
8mm 0° endoscope (470056)	○	○	○	○	○
8mm 30° endoscope (470057)	○	○	○	○	○
8mm monopolar curved scissors (hot shears) (470179)	○	○	○	○	○
8mm permanent cautery hook (470183)	○	○	○	○	○
8mm permanent cautery spatula (470184)	○	○	○	○	○
8mm micro bipolar forceps (471171)	○	○	○	○	○
8mm Maryland bipolar forceps (471172)	○	○	○	○	○
8mm fenestrated bipolar forceps (471205)	○	○	○	○	○
8mm curved bipolar dissector (471344)	○	○	○	○	○
8mm long bipolar grasper (471400)	○	○	○	○	○
8mm force bipolar (471405)	○	○	○	○	○
8mm small clip applier (470401)	○	○	○	○	○
8mm medium-large clip applier (470327)	○	○	○	○	○

8mm large clip applier (470230)	○	○	○	○	○
8mm large needle driver (471006)	○	○	○	○	○
8mm mega needle driver (470194)	○	○	○	○	○
8mm large SutureCut needle driver (471296)	○	○	○	○	○
8mm mega SutureCut needle driver (471309)	○	○	○	○	○
8mm tenaculum forceps (470207)	○	○	○	○	○
8mm small Graptor (grasping retractor) (470318)	○	○	○	○	○
8mm tip-up fenestrated grasper (470347)	○	○	○	○	○
8mm long tip forceps (471048)	○	○	○	○	○
8mm Cadiere forceps (471049)	○	○	○	○	○
8mm ProGrasp forceps (471093)	○	○	○	○	○
8mm cobra grasper (471190)	○	○	○	○	○
8mm Potts scissors (470001)	○	○	○	○	○
8mm round tip scissors (470007)	○	○	○	○	○
8mm black diamond micro forceps (470033)	○	○	○	○	○
8mm DeBakey forceps (470036)	○	○	○	○	○
8mm Resano forceps (470181)	○	○	○	○	○
8mm cardiac probe grasper (470215)	○	○	○	○	○
8mm atrial retractor short right (470246)	○	○	○	○	○
8mm dual blade retractor (470249)	○	○	○	○	○
8mm harmonic ACE curved shears (480275)	○	○	○	○	○
8mm EndoWrist suction irrigator (480299)	○	○	○	○	○
8mm Vessel Sealer Extend (480422)	○	○	○	○	○
8mm SynchroSeal (480440)	○	○	○	○	○
8mm SureForm 45 stapler (480445)	○	○	○	○	○
8mm SureForm 45 curved-tip stapler (480545)	○	○	○	○	○
8mm SureForm 60 stapler (480460)	○	○	○	○	○

8mm EndoWrist stapler 30 (470430)	◯	◯	◯	◯	◯
8mm EndoWrist stapler 30 curved-tip (470530)	◯	◯	◯	◯	◯
Other	◯	◯	◯	◯	◯

What type and quantity of other robotic instrument(s) were opened and USED?

____
(Please include item code(s))

Were any robotic instruments opened and UNUSED?

◯ Yes
◯ No

Type and Quantity of Robotic Instruments (Item Code) - Opened and UNUSED

	1	2	3	4	5
5mm curved scissors (478057)					
5mm permanent cautery hook (478090)					
5mm suction irrigator (478054)					
5mm Maryland bipolar forceps (478080)					
5mm fenestrated bipolar forceps (478093)					
5mm Cadiere forceps/grasper (478055)					
5mm fundus grasper (478058)					
5mm crocodile grasper (478059)					
5mm curved needle driver (478088)					
5mm wristed needle driver (478115)					
5mm Maryland dissector (478050)					
5mm medium-large clip applier (478053)					
8mm 0° endoscope (470056)					
8mm 30° endoscope (470057)					
8mm monopolar curved scissors (hot shears) (470179)					
8mm permanent cautery hook (470183)					
8mm permanent cautery spatula (470184)					
8mm micro bipolar forceps (471171)					
8mm Maryland bipolar forceps (471172)					
8mm fenestrated bipolar forceps (471205)					
8mm curved bipolar dissector (471344)					
8mm long bipolar grasper (471400)					
8mm force bipolar (471405)					
8mm small clip applier (470401)					
8mm medium-large clip applier (470327)					

8mm large clip applier (470230)	○	○	○	○	○
8mm large needle driver (471006)	○	○	○	○	○
8mm mega needle driver (470194)	○	○	○	○	○
8mm large SutureCut needle driver (471296)	○	○	○	○	○
8mm mega SutureCut needle driver (471309)	○	○	○	○	○
8mm tenaculum forceps (470207)	○	○	○	○	○
8mm small Graptor (grasping retractor) (470318)	○	○	○	○	○
8mm tip-up fenestrated grasper (470347)	○	○	○	○	○
8mm long tip forceps (471048)	○	○	○	○	○
8mm Cadiere forceps (471049)	○	○	○	○	○
8mm ProGrasp forceps (471093)	○	○	○	○	○
8mm cobra grasper (471190)	○	○	○	○	○
8mm Potts scissors (470001)	○	○	○	○	○
8mm round tip scissors (470007)	○	○	○	○	○
8mm black diamond micro forceps (470033)	○	○	○	○	○
8mm DeBakey forceps (470036)	○	○	○	○	○
8mm Resano forceps (470181)	○	○	○	○	○
8mm cardiac probe grasper (470215)	○	○	○	○	○
8mm atrial retractor short right (470246)	○	○	○	○	○
8mm dual blade retractor (470249)	○	○	○	○	○
8mm harmonic ACE curved shears (480275)	○	○	○	○	○
8mm EndoWrist suction irrigator (480299)	○	○	○	○	○
8mm Vessel Sealer Extend (480422)	○	○	○	○	○
8mm SynchroSeal (480440)	○	○	○	○	○
8mm SureForm 45 stapler (480445)	○	○	○	○	○
8mm SureForm 45 curved-tip stapler (480545)	○	○	○	○	○
8mm SureForm 60 stapler (480460)	○	○	○	○	○

8mm EndoWrist stapler 30 (470430)	◯	◯	◯	◯	◯
8mm EndoWrist stapler 30 curved-tip (470530)	◯	◯	◯	◯	◯
Other	◯	◯	◯	◯	◯

What type and quantity of other robotic instrument(s) were opened and UNUSED?

____
(Please provide item code(s))

Count of Operating Theatre Staff

	1	2	3	4	5
Surgical consultants	○	○	○	○	○
Surgical fellows	○	○	○	○	○
Surgical registrars/trainees	○	○	○	○	○
Anaesthetic consultants	○	○	○	○	○
Anaesthetic fellows	○	○	○	○	○
Anaesthetic registrars/trainees	○	○	○	○	○
Junior medical staff	○	○	○	○	○
Nurses	○	○	○	○	○
Theatre technicians	○	○	○	○	○
Patient services assistant/porter	○	○	○	○	○
Radiographer	○	○	○	○	○
Other	○	○	○	○	○

Who else was in the operating theatre?

____

Intraoperative Complications

Were there any non-robotic intraoperative complications?	◯ Yes<br>◯ No
Category of non-robotic intraoperative complication(s)	☐ ClassIntra I<br>☐ ClassIntra II<br>☐ ClassIntra III<br>☐ ClassIntra IV<br>☐ ClassIntra V
Description of non-robotic intraoperative complication(s) and how it affected the patient and/or the surgery	______________________________
Were there any robot-specific intraoperative complications?	◯ Yes<br>◯ No
Category of robot-specific intraoperative complication(s)	☐ Console-related malfunction<br>☐ Robotic instrument-related malfunction<br>☐ Conversion
Description of robot-specific intraoperative complication(s) and how it affected the patient and/or the surgery	______________________________
As an operating team, what reflections did you have about how the robotic surgery went?	______________________________
