# Supplementary material for: Advancing paediatric robotic surgery: building the foundation with databases
Source: J Robot Surg. 2026 Aug 12;20(1):832. doi: 10.1007/s11701-026-03774-y (PMC13468980; doi:10.1007/s11701-026-03774-y)
Supplement: Supplementary file 5 [file 11701_2026_3774_MOESM5_ESM.pdf]

Online Resource 5

REDCap Database Proforma
Part 3.1 - Postoperative Variables

Advancing Paediatric Robotic Surgery: Building the Foundation with Databases

Journal of Robotic Surgery

Calvin George Marangoly, Thomas Blanc, Mohan S. Gundeti, Craig A. Peters, Chris Kimber, Kiarash Taghavi

Corresponding author: Kiarash Taghavi, Department of Paediatric Urology, Monash Children's Hospital. Email: kiarash.taghavi@monash.edu

Postoperative Variables

Please complete the survey below.

Thank you!

[adm_type_plan] was the planned admission type for this patient. Did the actual admission type differ?	◯ Yes<br>◯ No
Actual admission type	◯ Elective day surgery<br>◯ Elective inpatient surgery<br>◯ Emergency
Analgesic requirements	☐ Nil<br>☐ Paracetamol<br>☐ NSAIDs<br>☐ Weak opioids (e.g. codeine, tramadol)<br>☐ Strong opioids (e.g. morphine, oxycodone, fentanyl)<br>☐ Gabapentinoids (e.g. gabapentin, pregabalin)<br>☐ Steroids<br>☐ Ketamine<br>☐ Other
Other analgesia	__________________________
Time to resumption of oral intake	__________________________<br>(Hours)
Were there any postoperative complications?	◯ Yes<br>◯ No
Category of postoperative complication(s)	☐ Clavien-Dindo Grade I<br>☐ Clavien-Dindo Grade II<br>☐ Clavien-Dindo Grade IIIa<br>☐ Clavien-Dindo Grade IIIb<br>☐ Clavien-Dindo Grade IVa<br>☐ Clavien-Dindo Grade IVb<br>☐ Clavien-Dindo Grade V
Description of postoperative complication(s) and how it affected the patient	__________________________________

Resource Consequences of Postoperative Complications

	Yes	No
Wound swab and culture	◯	◯
Urine dipstick/full ward test	◯	◯
Urinalysis	◯	◯
Blood tests	◯	◯
Blood crossmatch/group and hold	◯	◯
Blood culture	◯	◯
Ultrasound	◯	◯
X-ray	◯	◯
CT scan	◯	◯
MRI scan	◯	◯
IV fluids	◯	◯
Peripheral parenteral nutrition	◯	◯
Antibiotics	◯	◯
Simple analgesia (e.g. paracetamol, NSAIDs)	◯	◯
Opiate analgesia	◯	◯
Laxatives	◯	◯
Antiemetics	◯	◯
Blood products/transfusion	◯	◯
Other tests and/or therapies used	◯	◯

What other tests and/or therapies were used? ____________________________________

Was there an unplanned ICU admission?
◯ Yes
◯ No

ICU admission date ______________________________

Reason for ICU admission ____________________________________

Was there patient mortality while in ICU?
◯ Yes
◯ No

ICU discharge date ______________________________

Length of ICU stay ______________________________

Was there an unplanned return to theatre?
◯ Yes
◯ No

Return to theatre procedure	____
Return to theatre indication	____
Discharge date	________________________________
Length of hospital stay	________________________________
Was readmission required within 30 days of surgery?	◯ Yes<br>◯ No
Reason for readmission	____
Days alive and at home 30 days after surgery (DAH30)	________________________________<br>(Days)
Was there patient mortality within 30 days of surgery?	◯ Yes<br>◯ No
Was their death related to surgery?	◯ Yes<br>◯ No
