# Supplementary material for: Advancing paediatric robotic surgery: building the foundation with databases
Source: J Robot Surg. 2026 Aug 12;20(1):832. doi: 10.1007/s11701-026-03774-y (PMC13468980; doi:10.1007/s11701-026-03774-y)
Supplement: Supplementary file 6 [file 11701_2026_3774_MOESM6_ESM.pdf]

Online Resource 6

REDCap Database Proforma
Part 3.2 - CHU9D Postoperative

Advancing Paediatric Robotic Surgery: Building the Foundation with Databases

Journal of Robotic Surgery

Calvin George Marangoly, Thomas Blanc, Mohan S. Gundeti, Craig A. Peters, Chris Kimber, Kiarash Taghavi

Corresponding author: Kiarash Taghavi, Department of Paediatric Urology, Monash Children's Hospital. Email: kiarash.taghavi@monash.edu

CHU9D - Postoperative

Please complete the survey below.

Thank you!

Date of Completion ______________________________

Worried
- ◯ I don't feel worried today
- ◯ I feel a little bit worried today
- ◯ I feel a bit worried today
- ◯ I feel quite worried today
- ◯ I feel very worried today

Sad
- ◯ I don't feel sad today
- ◯ I feel a little bit sad today
- ◯ I feel a bit sad today
- ◯ I feel quite sad today
- ◯ I feel very sad today

Pain
- ◯ I don't have any pain today
- ◯ I have a little bit of pain today
- ◯ I have a bit of pain today
- ◯ I have quite a lot of pain today
- ◯ I have a lot of pain today

Tired
- ◯ I don't feel tired today
- ◯ I feel a little bit tired today
- ◯ I feel a bit tired today
- ◯ I feel quite tired today
- ◯ I feel very tired today

Annoyed
- ◯ I don't feel annoyed today
- ◯ I feel a little bit annoyed today
- ◯ I feel a bit annoyed today
- ◯ I feel quite annoyed today
- ◯ I feel very annoyed today

Schoolwork/Homework (such as reading, writing, doing lessons)
- ◯ I have no problems with my schoolwork/homework today
- ◯ I have a few problems with my schoolwork/homework today
- ◯ I have some problems with my schoolwork/homework today
- ◯ I have many problems with my schoolwork/homework today
- ◯ I can't do my schoolwork/homework today

Sleep
- ◯ Last night I had no problems sleeping
- ◯ Last night I had a few problems sleeping
- ◯ Last night I had some problems sleeping
- ◯ Last night I had many problems sleeping
- ◯ Last night I couldn't sleep at all

Daily routine (things like eating, having a bath/shower, getting dressed)
- ◯ I have no problems with my daily routine today
- ◯ I have a few problems with my daily routine today
- ◯ I have some problems with my daily routine today
- ◯ I have many problems with my daily routine today
- ◯ I can't do my daily routine today

Able to join in activities (things like playing out with your friends, doing sports, joining in things)	◯ I can join in with any activities today<br>◯ I can join in with most activities today<br>◯ I can join in with some activities today<br>◯ I can join in with a few activities today<br>◯ I can join in with no activities today
