# Supplementary material for: Advancing paediatric robotic surgery: building the foundation with databases
Source: J Robot Surg. 2026 Aug 12;20(1):832. doi: 10.1007/s11701-026-03774-y (PMC13468980; doi:10.1007/s11701-026-03774-y)
Supplement: Supplementary file 7 [file 11701_2026_3774_MOESM7_ESM.pdf]

Online Resource 7

REDCap Database Proforma
Part 3.3 - AHPEQS

Advancing Paediatric Robotic Surgery: Building the Foundation with Databases

Journal of Robotic Surgery

Calvin George Marangoly, Thomas Blanc, Mohan S. Gundeti, Craig A. Peters, Chris Kimber, Kiarash Taghavi

Corresponding author: Kiarash Taghavi, Department of Paediatric Urology, Monash Children’s Hospital. Email: kiarash.taghavi@monash.edu

Australian Hospital Patient Experience Questionnaire Set (AHPEQS)

Please complete the survey below.

Thank you!

Date of completion	______________________________
My views and concerns were listed to	◯ Always<br>◯ Mostly<br>◯ Sometimes<br>◯ Rarely<br>◯ Never<br>◯ Didn't apply
My individual needs were met	◯ Always<br>◯ Mostly<br>◯ Sometimes<br>◯ Rarely<br>◯ Never
When a need could not be met, staff explained why	◯ Always<br>◯ Mostly<br>◯ Sometimes<br>◯ Rarely<br>◯ Never
I felt cared for	◯ Always<br>◯ Mostly<br>◯ Sometimes<br>◯ Rarely<br>◯ Never
I was involved as much as I wanted in making decisions about my treatment and care	◯ Always<br>◯ Mostly<br>◯ Sometimes<br>◯ Rarely<br>◯ Never
I was kept informed as much as I wanted about my treatment and care	◯ Always<br>◯ Mostly<br>◯ Sometimes<br>◯ Rarely<br>◯ Never
It was clear to me that staff had communicated with each other about my treatment and care	◯ Always<br>◯ Mostly<br>◯ Sometimes<br>◯ Rarely<br>◯ Never<br>◯ Didn't apply

I received pain relief that met my needs	◯ Always<br>◯ Mostly<br>◯ Sometimes<br>◯ Rarely<br>◯ Never<br>◯ Didn't apply
When I was in hospital I felt confident in the safety of my treatment and care	◯ Always<br>◯ Mostly<br>◯ Sometimes<br>◯ Rarely<br>◯ Never
I experienced unexpected harm or distress as a result of my treatment and care	◯ Yes, physical harm<br>◯ Yes, emotional distress<br>◯ Yes, both<br>◯ No
My harm or distress was discussed with me by staff	◯ Yes<br>◯ No<br>◯ Not sure<br>◯ Didn't want to discuss it
Overall, the quality of treatment and care I received was	◯ Very good<br>◯ Good<br>◯ Average<br>◯ Poor<br>◯ Very poor
